# Zoster Sine Herpete: An Unusual Presentation

**DOI:** 10.7759/cureus.91119

**Published:** 2025-08-27

**Authors:** Abdelnassir Abdelgabar, Ayesha Farooq, Quratulain Jabbar, Mohammed Elsayed

**Affiliations:** 1 General Internal Medicine and Acute Medicine, Diana, Princess of Wales Hospital, Grimsby, GBR

**Keywords:** acyclovir, meningoencephalitis, thunderclap headache, varicella, zoster sine herpete

## Abstract

Varicella zoster virus (VZV) commonly causes chicken pox, following which the virus becomes latent in the ganglionic neurons across the entire neuroaxis. Reactivation of VZV typically causes herpes zoster (shingles), with its characteristic dermatomal distribution and post-herpetic neuralgia, as its most common complication. In some rare cases, reactivation of VZV may occur in the absence of the typical skin manifestations, a condition known as zoster sine herpete (ZSH), making the diagnosis of VZV infection extremely difficult, particularly when it presents in the form of one of the rare complications, such as encephalitis, as in the case of our patient. In this case report, we present a case of ZSH in an immunocompetent lady presenting with brief thunderclap headache (TCH) and confusion with no prior history of shingles. The diagnosis was confirmed with the positive polymerase chain reaction (PCR) for VZV with clinical and biochemical cerebrospinal fluid (CSF) resolution following a two-week treatment with intravenous acyclovir, substantiating the diagnosis of ZSH.

## Introduction

Varicella zoster virus (VZV) is an exclusively human herpes virus that causes chicken pox (varicella) as an acute primary infection, usually in children. Following this, VZV becomes latent, existing in noninfectious forms in the dorsal root ganglion (DRG), enteric ganglia, autonomic ganglia, and cranial and peripheral nerve ganglia across the whole neuroaxis [1,2]. The latent VZV may reactivate many years or even decades later, resulting in several varieties of presentations [3]. The most common of these protean presentations is shingles, a painful pruritic skin eruption that occurs in characteristic dermatomal distribution [3]. Reactivation is mainly triggered by factors compromising cell-mediated immunity, such as advanced age, malignancy, immunosuppressive medications, and even psychological stress [3]. Once shingles appear, even many decades later after chicken pox, the diagnosis of herpes zoster (HZV) becomes easily established. However, VZV reactivation can occur in areas that do not project to the skin, such as the enteric nervous system, autonomic nervous system, and spinal cord or central nervous system, resulting in serious variable presentations in the absence of the typical skin manifestations. This presentation, termed ZSH, is likely to be misdiagnosed or attributed to other pathologies, leading to delayed or no treatment with serious fatal sequelae like stroke, encephalitis, and cerebral vasculopathy [4,5].

## Case presentation

A 65-year-old female with a past medical history of childhood chicken pox and stable angina presented to the accident and emergency (A&E) with a sudden, brief, violent frontal headache that she had never experienced before. It was associated with vomiting twice and very brief lower neck and back pain, with no radiation to the legs. There was no photophobia, rash, fever, or neck stiffness, and no preceding flu-like symptoms. Patient went to work the next day after her headache completely subsided, but returned home as she was noticed to be slow, clumsy, and mildly confused. Husband also agreed that she was not her usual self. In A&E, her vital signs were normal, and she was not in apparent pain or distress. There was no rash or signs of meningism. Glasgow coma scale (GCS) was 15/15, and the patient was not confused. There were no sensory or focal neurological deficits. Gait was normal, and no cerebellar signs were present. The patient was admitted to rule out subarachnoid hemorrhage, intracranial hemorrhage, or encephalitis. Because of the possibility of encephalitis, the patient was started on both acyclovir and ceftriaxone pending lumbar puncture (LP).

**Figure 1:**
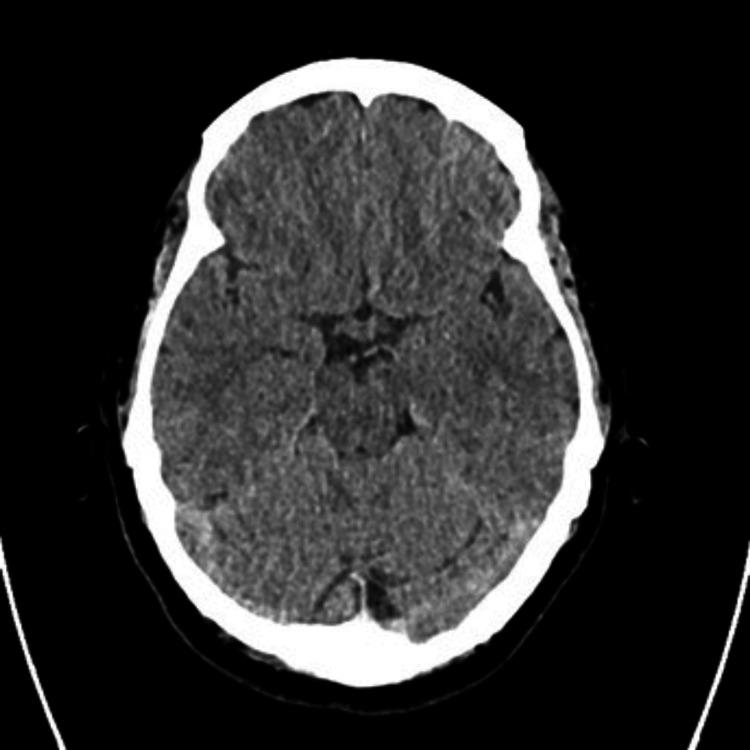
Normal noncontrast CT scan of the brain This figure shows a CT scan of the brain performed to rule out SAH, which was normal. SAH: Subarachnoid hemorrhage.

**Figure 2:**
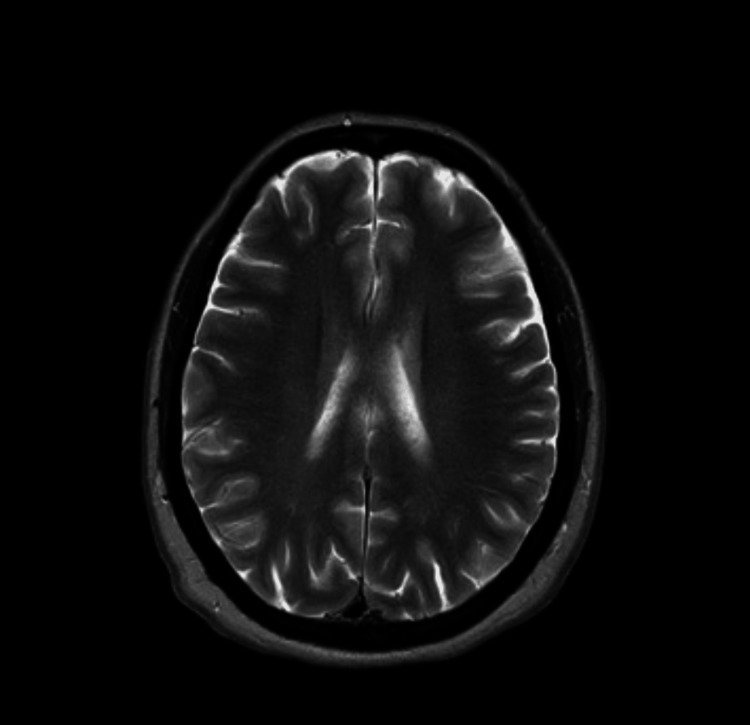
Normal MRI scan of the brain A brain MRI was done to rule out encephalitis and SAH. SAH: Subarachnoid hemorrhage.

All basic investigations including full blood count (FBC), renal and liver function, blood sugar, and CRP (C-reactive protein) were all within normal limits. Noncontrast computerized tomography scan (CT) of the brain was normal (Figure 1). This was followed by magnetic resonance imaging (MRI) of the brain, which was also normal (Figure 2).

In view of the cerebrospinal fluid (CSF) findings (Table 1), the diagnosis of VZV encephalitis was considered, ceftriaxone was stopped, and acyclovir was continued. It was also felt necessary to rule out myelitis because of the history of brief neck and lower back pain, in light of the positive VZV polymerase chain reaction (PCR).

**Table 1:** CSF findings before and after acyclovir treatment CSF findings, both before and after treatment, confirmed the response to acyclovir treatment.

CSF findings	Before treatment	After treatment	Reference range
Appearance	Clear colorless	Clear colorless	Clear
Volume, ml	0.5	0.25	
White blood cells, /mm³	499	49	0-5 (mostly lymphocytes)
Percentage of polymorphs	0%	5%	
Percentage of lymphocytes	100%	95%	
Red blood cells, /mm³	6	3	<5
Gram stain	Organism not seen	Organism not seen	
CSF protein, mg/L	1363	450	150-450
CSF glucose, mmol/L	3.3	4.2	2.2-4.4
CSF lactate µ/L	33	24	<40
CSF xanthochromia	No evidence to support SAH		
Enterovirus by PCR	Not detected	Not detected	
Herpes simplex virus by PCR	Not detected	Not detected	
Parechovirus by PCR	Not detected	Not detected	
Varicella zoster by PCR	Detected	Not detected	

**Figure 3:**
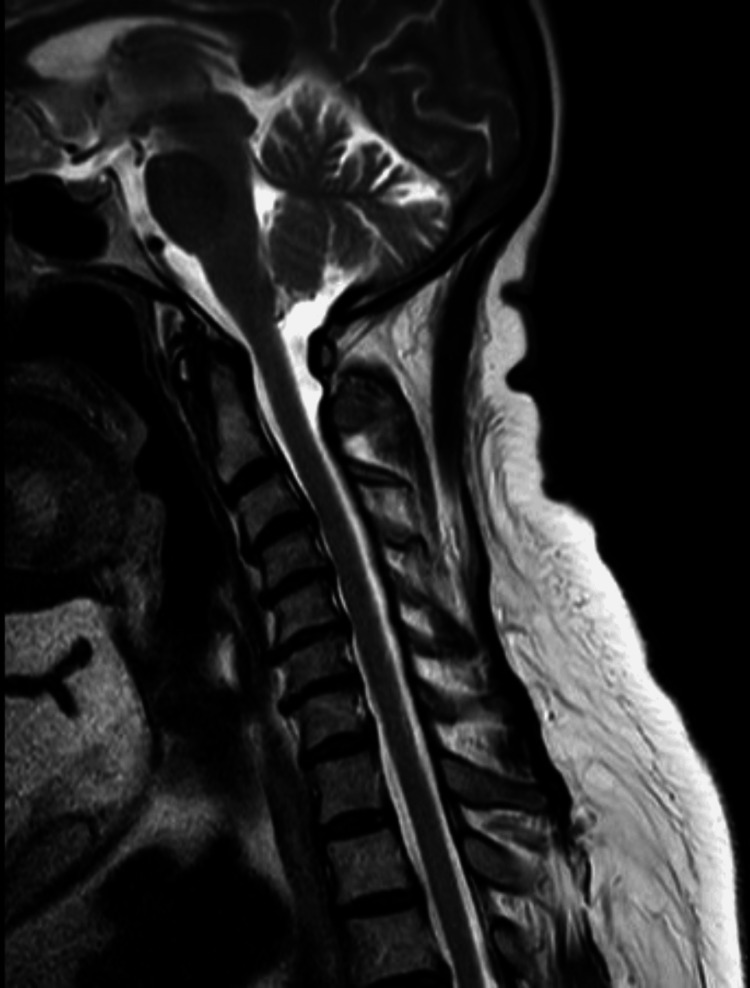
MRI of the cervical spine MRI of the C-spine was done to rule out cervical myelitis as the patient was complaining of lower neck pain.

**Figure 4:**
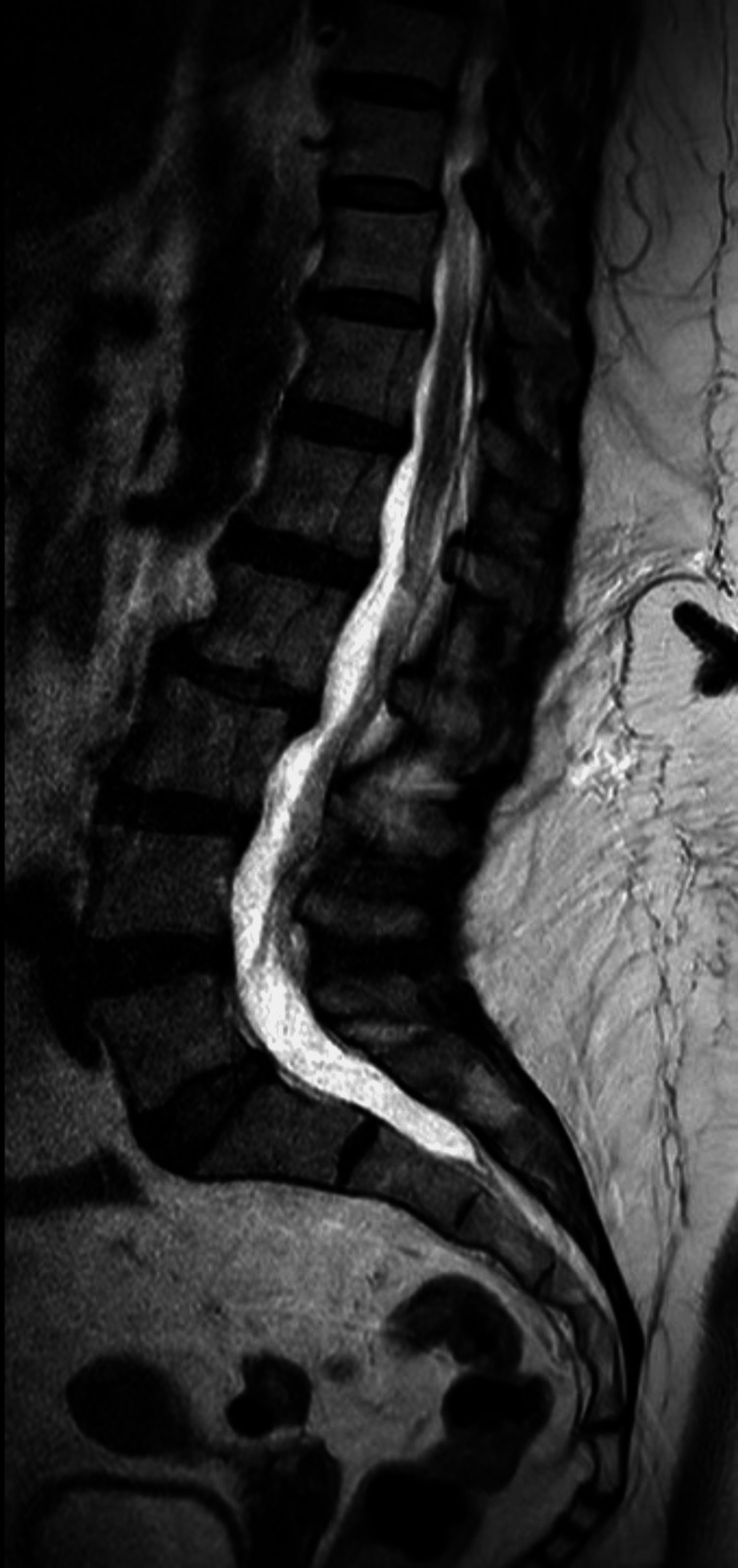
MRI of the lumbosacral spine showing no evidence of myelitis MRI of the lumbosacral spine was also done to rule out myelitis as the patient was complaining of lower back pain.

MRI cervical spine (Figure 3) and MRI lumbosacral spine (Figure 4) were both normal. After two weeks of intravenous acyclovir, LP was repeated (Table 1). Acyclovir was stopped on the advice of the microbiologist due to the clearance of VZV from the CSF on PCR with normalization of the CSF protein and reduction of the lymphocytes, indicating excellent response to acyclovir. The patient herself remained asymptomatic throughout her hospital stay.

## Discussion

ZSH is one of the atypical clinical manifestations of VZV reactivation. It usually presents as neuropathic pain in the absence of the hallmark of the antecedent rash associated with HZ (shingles) [6]. The pain is usually dermatomal often affecting the thoracic or cranial dermatomes, with the trigeminal and facial nerves the most commonly affected. The pain can present in different forms such as allodynia, paresthesia, dysesthesia, numbness, or tingling. ZSH patients experience more severe pain of higher intensity and longer duration, requiring a wider range of drugs compared to HZ patients [7]. The lack of the characteristic VZV rash can lead to missed or delayed diagnosis of ZSH with serious central nervous system complications such as encephalitis, myelitis, meningitis, or stroke [8].

ZSH is most likely underreported and underdiagnosed as clinical diagnosis is difficult and the confirmatory tests are not part of the routine laboratory workup. No studies have investigated the prevalence of ZSH; however, extrapolating from the high positivity of VZV IgG serologies in young adults in the United States and the common pathophysiology between HZ and ZSH, the incidence of the latter must be high [9,10].

Although HZ is generally diagnosed on clinical grounds alone, the diagnosis of ZSH is made extremely challenging due to the lack of a characteristic rash. Therefore, high clinical suspicion is needed, particularly in patients with unilateral neuropathic pain, peripheral or cranial nerve palsies, or muscle paralysis without rash, when other causes are ruled out [11]. For the diagnosis of ZSH, it is important to find evidence of VZV reactivation based on laboratory investigation [7].

The two most common approaches to detect VZV reactivation currently in practice are VZV DNA detection by PCR in CSF, serum, or saliva - most commonly from CSF, which can also be tested for protein, cells, and Gram stain to help rule out other diagnoses as well. The other commonly used method is the detection of anti-VZV IgG and anti-VZV IgM. While the PCR is highly sensitive and specific, anti-VZV antibodies are time-dependent and can give false-negative results [12].

Our patient is unique in her presentation, which defied many common features of HZV and even ZSH. She was young, immunocompetent, and had no prodromal symptoms or fever. She lacked the classical rash or the neuropathic dermatomal pain. She also had a brief nonrecurring thunderclap headache (TCH), which is an extremely rare manifestation of ZSH. To date, there have been very few case reports of TCH with ZSH, almost all due to vasculopathy, presenting as reversible cerebral vasoconstriction, stroke, or subarachnoid hemorrhage (SAH), but none due to encephalitis [13]. TCH in ZSH without vasculopathy is virtually unheard of. Our case also highlights the possible presentation of ZSH encephalitis as a brief TCH; therefore, such a presentation, albeit brief, should alarm clinicians to look for ZSH in addition to the other known serious causes.

Take-home message

TCH in the absence of the known causes such as SAH, RCVC, and ICH should prompt consideration of VZV-related meningitis or encephalitis. High clinical suspicion of ZSH is needed for early diagnosis and hence avoidance of serious long-term complications. Virological confirmation via CSF PCR is crucial in diagnosing VZV encephalitis, especially when typical features like rash are absent.

## Conclusions

This report illustrates the diagnostic challenges posed by ZSH, especially in immunocompetent individuals who present without the classic shingles rash or typical prodromal symptoms such as fever, malaise, and flu-like symptoms. Our patient primarily exhibited TCH and lacked dermatological manifestations, initially suggesting SAH as the most likely diagnosis. However, virological confirmation through PCR was crucial in establishing ZSH encephalitis. The patient’s complete clinical and biochemical recovery following intravenous acyclovir further supports the diagnosis. This case emphasizes the importance of including VZV in the differential diagnosis of unexplained neurological symptoms, even when rash is absent, and demonstrates the important role of early antiviral treatment in preventing severe complications like encephalitis and myelitis. Moreover, it draws attention to considering TCH within the differential diagnosis of VZV encephalitis and emphasizes the need for heightened clinical suspicion to diagnose ZSH, which can be difficult to identify based solely on clinical presentation.
